# Effect of stress urinary incontinence on vaginal microbial communities

**DOI:** 10.1186/s12866-024-03237-0

**Published:** 2024-04-04

**Authors:** Man Zhang, Yanhua Zhou, Siqi Yao, Yiming Zhao, Syeda Sundas Batool, Jing Huang, Li Jiang, Dayu Yan, Wenguang Yan, Zheng Yu

**Affiliations:** 1 Human Microbiome and Health Group, Department of Microbiology, School of Basic Medical Science, Central South University, Changsha, Hunan China; 2 Department of Rehabilitation Medicine, The Third Xiangya Hospital, Central South University, Changsha, China; 3 Department of Parasitology, School of Basic Medical Science, Central South University, Changsha, Hunan China; 4 Department of Gynecology, The Third Xiangya Hospital, Central South University, Changsha, China

**Keywords:** Stress urinary incontinence, Vaginal microbiota, Dysbiosis, *Lactobacillus*

## Abstract

**Background:**

Postpartum women often experience stress urinary incontinence (SUI) and vaginal microbial dysbiosis, which seriously affect women’s physical and mental health. Understanding the relationship between SUI and vaginal microbiota composition may help to prevent vaginal diseases, but research on the potential association between these conditions is limited.

**Results:**

This study employed 16S rRNA gene sequencing to explore the association between SUI and vaginal dysbiosis. In terms of the vaginal microbiota, both species richness and evenness were significantly higher in the SUI group. Additionally, the results of NMDS and species composition indicated that there were differences in the composition of the vaginal microbiota between the two groups. Specifically, compared to postpartum women without SUI (Non-SUI), the relative abundance of bacteria associated with bacterial dysbiosis, such as *Streptococcus*, *Prevotella*, *Dialister*, and *Veillonella*, showed an increase, while the relative abundance of *Lactobacillus* decreased in SUI patients. Furthermore, the vaginal microbial co-occurrence network of SUI patients displayed higher connectivity, complexity, and clustering.

**Conclusion:**

The study highlights the role of *Lactobacillus* in maintaining vaginal microbial homeostasis. It found a correlation between SUI and vaginal microbiota, indicating an increased risk of vaginal dysbiosis. The findings could enhance our understanding of the relationship between SUI and vaginal dysbiosis in postpartum women, providing valuable insights for preventing bacterial vaginal diseases and improving women’s health.

**Supplementary Information:**

The online version contains supplementary material available at 10.1186/s12866-024-03237-0.

## Introduction

Stress urinary incontinence (SUI) is the involuntary leakage of urine due to increased bladder pressure during body movements like coughing, squatting, or running. SUI is prevalent in postpartum and older women, with a prevalence of 18–34% according to studies [1]. Postpartum SUI can potentially cause depressive symptoms and exacerbate the patient’s mental health damage [2]. According to statistics, urinary incontinence affects millions of people worldwide and is recognized as a major health problem with a significant social and economic burden by the World Health Organization [3]. Therefore, the study of urinary incontinence complications and their connection is crucial.

Currently, the research on the urinary microbiota has made great progress. Especially in the relationship between the urinary microbiota and reproductive tract diseases and the interaction with the human body [4]. The common clinical belief used to be that healthy urine is sterile, and the presence of bacteria associated with an inflammatory response in urine indicated a urinary tract infection. However, several studies have reported the presence of bacteria in the urine microbiota of health individuals, indicating that it is not completely sterile [5, 6]. In fact, research indicated that the urine microbiota of women is predominantly composed of *Lactobacillus*, while in men, *Corynebacterium* is more prevalent [7]. In addition, previous studies have demonstrated a robust connection between urogenital diseases and vaginal bacteria [8–10].

Female vaginal microbiota is a key factor in maintaining vaginal health. The reproductive tract microbiota of healthy women is predominantly composed of *Lactobacillus*, and the vaginal microbial diversity is relatively low [11]. The increase in vaginal microbial diversity and the significant decrease in the relative abundance of *Lactobacillus* imply a disruption of the vaginal microbiota, thereby increasing the risk of vaginal diseases in women [12]. Healthy women’s vaginal microenvironment exhibits self-regulation due to *Lactobacillus* secreting substances like lactic acid, hydrogen peroxide, and bacteriocins, protecting it from pathogenic bacteria infection [13]. On the other hand, when pathogenic bacteria invade, lactic acid bacteria in the vagina play an anti-inflammatory role, stimulating the host’s immune response and resisting the invasion of pathogenic bacteria [14]. The most common forms of vaginal infections are bacterial vaginosis (BV), vulvovaginal candidiasis (VC), and aerobic vaginitis (AV) [15].

Importantly, various types of *Lactobacillus* play a crucial role in preventing microbial imbalance, inhibiting pathogen adhesion, and colonizing vaginal epithelium. For example, different strains of vaginal probiotics, such as *Lactiplantibacillus plantarum* (formerly *Lactobacillus plantarum*), *Lactobacillus gasseri*, and *Lactobacillus acidophilus*, can individually contribute to maintaining a balanced vaginal microbiota. Besides, when these bacteria coexist, their functions may overlap, particularly in cases of BV and AV [16, 17]. The main species of *Lactobacillus* present in the vaginal microbiota are *Lactobacillus iners* and *Lactobacillus crispatus* [18]. *Lactobacillus crispatus* is associated with lower vaginal pH and a more stable pregnancy, with the lower pH aiding in suppressing dysbiosis and promoting anti-inflammatory and antibacterial effects [12]. In contrast, *Lactobacillus iners* does not produce H_2_O_2_, has lower efficiency in producing D-lactic acid. The role of *Lactobacillus iners* in maintaining vaginal health is still not clear [19]. In certain conditions, an imbalance in the vaginal microbiota may be caused by medication use, unhealthy lifestyle choices, or hormonal changes during pregnancy, which can disrupt the temperature, humidity, pH balance, and protective barrier of the vaginal environment, allowing pathogenic bacteria to thrive and cause infection [14]. Given the anatomical proximity between the female urethra and vagina, it is widely acknowledged that there exists a close relationship between the microbiota of these two regions [20]. Therefore, it is still worthwhile to further explore the relationship between urological diseases and vaginal microbiota composition, as well as the impact of SUI on vaginal microbiota composition.

In this study, vaginal discharge samples from postpartum women with and without SUI were collected. We aimed to investigate the impact of SUI on the vaginal dysbiosis using 16S rRNA gene sequencing. Our study contributes to better understanding the association between the occurrence of SUI and vaginal diseases and provides new insights for the prevention of vaginal diseases caused by SUI.

## Materials and methods

### Sample collection

To investigate the relationship between SUI and vaginal microbiota in postpartum women, we recruited volunteers at the Third Xiangya Hospital in Changsha, Hunan Province, China, and collected vaginal discharge samples from 32 postpartum women. The study was approved by the Ethics Committee of the Third Xiangya Hospital of Central South University and conducted in accordance with the relevant guidelines and regulations (IRB No. 22,133). Written informed consent was obtained from the participants before the study, and all samples and questionnaires were voluntary. Patients with diabetes were excluded, as well as those who had engaged in sexual activity during the previous two days or had used antibiotics within the previous three months. Postpartum women were split into two groups based on their SUI status: individuals without SUI (Non-SUI, *n* = 19) and subjects with SUI (SUI, *n* = 13), and the demographic characteristics of participants were shown in **Supplementary Table 1**. SUI was diagnosed based on self-reporting, medical reports, and an evaluation of pelvic floor muscle strength and function using the Aa, Ap, TVL, C, D, P, E, R, F, and I indexes.

Samples of vaginal discharge were obtained using sterile swabs. The subject laid on an examination table and a vaginal speculum was inserted to visualize the cervix. The sterile swab was carefully inserted into the vagina and rotated to ensure that there was sufficient discharge from the vaginal walls. The swab was placed in a sterile tube for transport to the laboratory. All samples were stored at -80 °C until further processing.

### Polymerase chain reaction and high-throughput sequencing of 16S rRNA gene

Total genomic DNA from vaginal secretion samples was extracted by OMEGA Soil DNA Kit (M5636-02) (Omega Bio-Tek, Norcross, GA, USA). Total genomic DNA was quantified by Nanodrop NC2000 (Thermo Fisher Scientfic, Waltham, MA, USA) for DNA quantification, and the quality of DNA extraction was examined by 1.2% agarose gel electrophoresis. Next, polymerase chain reaction (PCR) amplification and high-throughput sequencing of the V3-V4 region of the 16S rRNA gene were performed using forward primer 27F (5′-AGAGTTTGATCMTGGTCTCAG-3′) and reverse primer 1492R (5′-GGTTACCTTGTACTTT-3′) for PCR amplification. The qualified libraries were sequenced on the Illumina NovaSeq platform with the NovaSeq-PE250 sequencing strategy at Shanghai Personal Biotechnology Co., Ltd. (Shanghai, China). The sequencing length was 300 bp, and all samples had Q20 values greater than 98% and Q30 values greater than 95%. The average number of reads for all samples was 90,475, with a minimum of 71,864 and a maximum of 138,129 reads. After sequencing, the sequencing data was matched with the corresponding samples based on barcodes. Subsequently, the sequences were imported into Quantitative Insights into Microbial Ecology (QIIME2, 2021.2) software for quality control and denoising to obtain clean reads [21, 22]. Then, Deblur was used to perform dereplication of the sequence data and create feature tables and feature representative sequences [23]. After generating the feature table and feature representative sequences, based on a 99% sequence similarity threshold, the data was combined and clustered into operational taxonomic units (OTUs) [22]. Species annotation was performed on the sequence based on Silva 138 reference sequence and plugin feature classifier for further visualization and analysis [24].

### Data analysis

The data analysis in this study was based on the R language (v.4.2.3) [25]. Alpha diversity analysis was used to assess the species richness and evenness of the microbial community in this habitat. Alpha diversity indices calculated in this analysis included the Gini-Simpson index and Pielou evenness index. The calculation was done using the diversity function of the vegan package (v.2.6-4) [26].

Beta diversity was used to assess differences in microbial community composition between the SUI and Non-SUI groups. The diversity index of vaginal microbial communities was calculated using “vegdist” function based on Bray-Curtis distance after data extraction. Non-metric multidimensional scaling (NMDS) was performed using the “metaMDS” function in the vegan package (v.2.6-4), with a stress value less than 0.2 indicating a good fit of the model [27, 28].

For microbial community composition, the species were ranked based on their total relative abundance across all samples, and the top ten species in terms of relative abundance were displayed. Then the visualization was completed using the ggbarplot function in the ggpubr package (v.0.6.0). Next, the UpsetR package (v.1.4.0) was used to plot the Upset plot to show the distribution of the top 10 bacteria in terms of abundance in different samples. Genus level Manhattan plot computed using the edgeR package (v.3.42.4) based on taxonomy information (FDR ≤ 0.05). In addition, the Kruskal-Wallis test was used to compare the differences in relative abundance of different species between groups and was plotted using ggboxplot function in the ggpubr package (v.0.6.0).

Besides, Linear Discriminant Analysis (LDA) Effect Size (LEfSe) was used to screen biomarkers between groups, and the threshold was set at *P* < 0.05 and LDA ≥ 4 [29]. Finally, based on the Spearman correlation coefficient calculation, two sets of co-occurrence network matrices were established. Then, we corrected for multiple P-value tests using the False Discovery Rate by Benjamini-Hochberg (FDR-BH) method. Modules were divided according to the connectivity of the modules. Spearman correlation coefficients and corrected *P*-values were 0.6 and 0.05. Finally, the co-occurrence networks were visualized in Gephi software (v.0.9.5) [30]. The correlation between species was calculated using the Pearson correlation coefficient, and the correlation matrix was visualized using the corrplot package (v.0.92).

SPSS (v.25.0) was used to calculate statistical differences between baseline data for groups Non-SUI and SUI. Consecutive variables were tested for differences between groups using the Mann-Whitney U test with mean and range (min-max) or mean standard deviation (SD) displayed, and *P* < 0.05 was considered a statistically significant difference between groups. For categorical data, descriptive statistics were presented using numbers and percentages, and Fisher’s exact test was used for analysis.

## Results

### Clinical characteristics of the study subjects

The demographic characteristics of participants in the SUI (case group, *n* = 13) and Non-SUI (control group, *n* = 19) groups indicate: no significant differences were observed between the case group and the control group in terms of age, gestation, partitioning, weight gain, and birthweight. However, it was worth noting that a marginal difference in BMI was detected between the two groups (*P* = 0.03) (Supplementary Table 1).

### The diversity of the vaginal microbiota

**Fig. 1:**
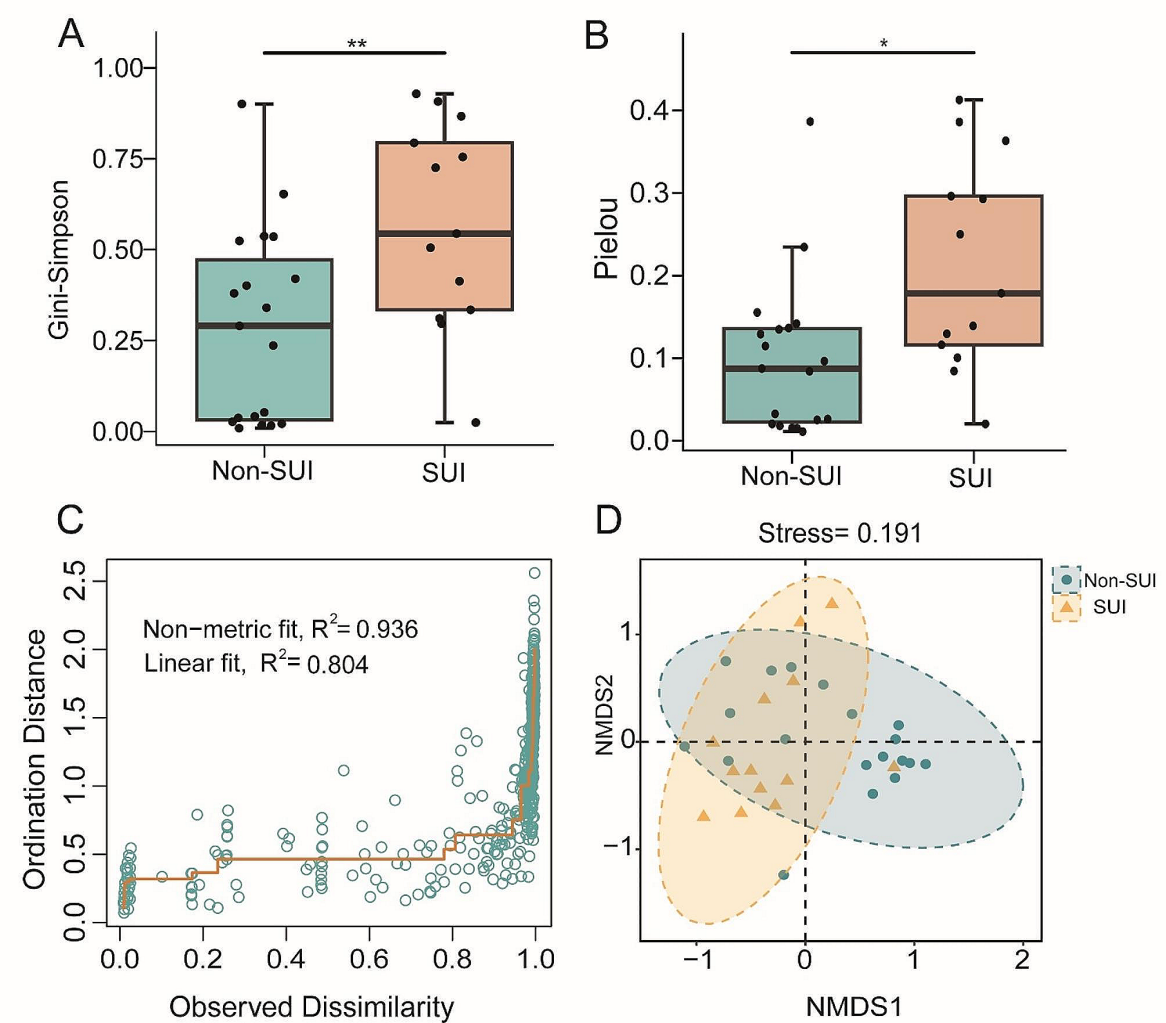
Diversity analysis of vaginal microbial community. Alpha diversity index analysis (**A**) Gini-Simpson (**P < 0.01). (**B**) Pielou evenness index (*P < 0.05). Beta diversity analysis (**C**) Shepard plot of dimensional reduction results for sample data, basically all points are concentrated near the line segment, indicating a small discrepancy between the reduced distances and actual distances, the dimensional reduction results exhibit high accuracy. (**D**) NMDS analysis (Stress < 0.2), the ellipse contains 95% of the samples in each group.

Dilution curves were computed and recorded after five random samples, with a minimum sampling depth of 50,000. As sampling depth increased, dilution curves leveled off, indicating reasonable sequencing data coverage (Supplementary Fig. 1). For the alpha diversity indexes calculated in this study, the Gini-Simpson index (*P* < 0.01) (Fig. 1A) and Pielou (*P* < 0.05) (Fig. 1B) were significantly higher in group SUI compared to Non-SUI, indicating that the vaginal microbial community composition of patients with SUI was more complex and diverse. The NMDS analysis effectively represented microbial communities, with differences in vaginal microbiota between groups and stress = 0.191 (< 0.2) (Fig. 1C, D).

### The composition of the vaginal microbiota

**Fig. 2:**
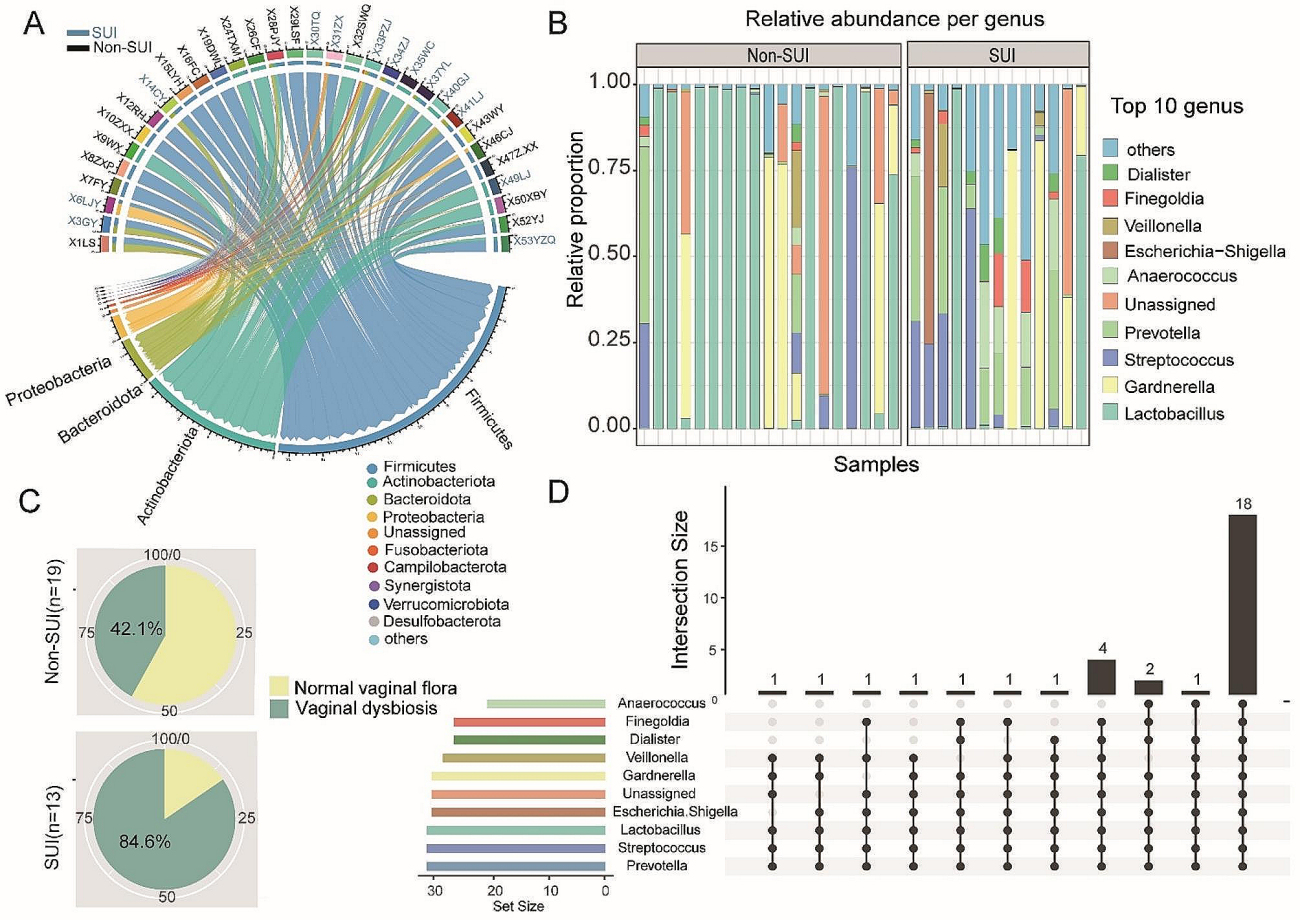
The relative abundances of taxonomy at the phylum level and genus level. (**A**) Microbial community composition at the phylum level of all samples, with the blue font indicating samples from the SUI group and the black font indicating samples from the Non-SUI group. (**B**) The relative abundance of microbial community composition at the genus level in group Non-SUI and SUI. (**C**) Proportion of dysbacteriosis in samples of group Non-SUI and SUI. (**D**) Upset diagram shows the distribution of the top ten bacteria in all samples at the genus level, with black columns indicating the number of samples and black dots indicating the existence of the bacteria.

At the phylum level, the microbial community composition of both groups was dominated by *Firmicutes*, *Actinobacteriota*, *Bacteroidota*, and *Proteobacteria*. In comparison to the SUI group, the relative abundance of *Bacteroidetes* was higher in the Non-SUI group (Fig. 2A). At the genus level, most of the samples in the Non-SUI group were still dominated by *Lactobacillus* (57.9%). But in the SUI group, the microbial community composition of most samples was no longer dominated by *Lactobacillus* but showed increased relative abundance of other bacteria such as *Prevotella*, *Gardnerella*, and *Streptococcus*, indicating the occurrence of microbial dysbiosis (Fig. 2B). Based on the proportion of samples with dysbiosis of the vaginal microbiota in two groups (whether the vaginal microbiota is dominated by *Lactobacillus*), the statistical results showed 42.1% (*n* = 8) of the Non-SUI group (*n* = 19) and 84.6% (*n* = 11) of the SUI group (*n* = 13) had dysbiosis (Fig. 2C). The results of logistic regression analysis showed a statistically significant association between SUI and vaginal dysbiosis (*P* = 0.016, OR = 82.977) (Supplementary Table 2). The upset analysis revealed the presence of certain bacterial genera in the samples. Specifically, *Lactobacillus*, *Prevotella*, and *Streptococcus* were present in all samples, indicating their high abundance across the dataset. Additionally, the *Gardnerella* was detected in 31 samples, suggesting its relatively common occurrence. The *Anaerococcus* was observed in 23 samples, indicating its presence in a substantial number of samples as well (Fig. 2D).

### Screening biomarkers for the sample groups

**Fig. 3:**
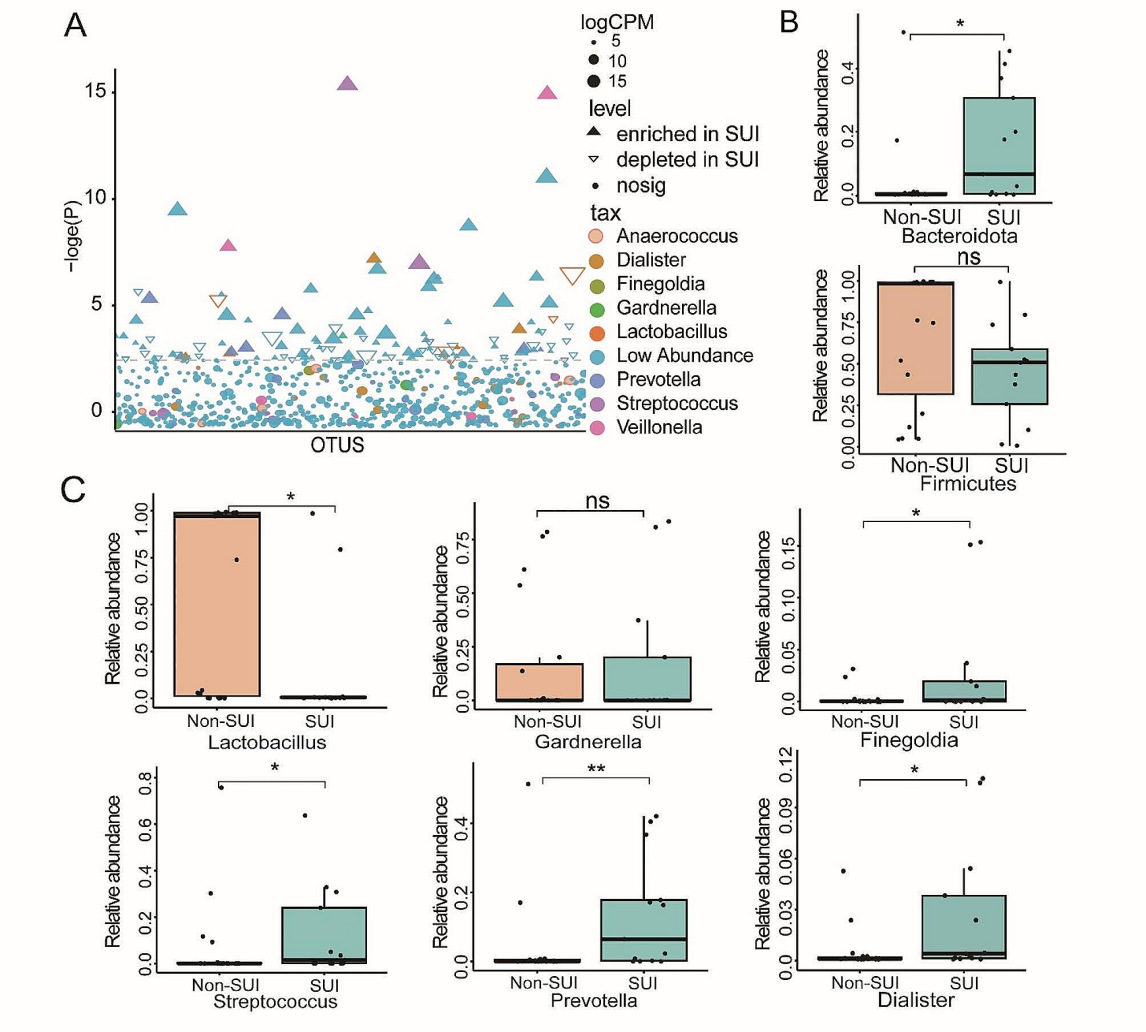
Differential analysis of species relative abundance at phylum and genus level. (**A**) The Manhattan plot compares Non-SUI and SUI groups using OTUs and p-values obtained through natural logarithmic transformation. The node size represents relative abundance of OTUs, while CPM (Count Per Million) represents fractions of a million. Different colors represent different genera, and the shape of the node indicates the type of change, whether up-regulated enriched (positive solid triangle), down-regulated depleted (inverted hollow triangle), or no significant difference change (solid nodes). Differences abundance at the phylum and genus level among two groups (Wilcox. test, *P < 0.05, ** *P* < 0.01). (**B**) Phylum level: *Bacteroidota*, *Firmicutes* (**C**) Genus level: *Streptococcus*, *Prevotella*, *Dialister*, *Veillonlla.*

To elucidate the bacterial differences among the groups, we conducted an analysis on the variation in bacterial abundance between the Non-SUI and SUI groups and visualized the differences using a Manhattan plot. In comparison with the Non-SUI group, the increased relative abundance of bacterial species was greater than the decreased relative abundance, and the number and diversity of significantly enriched OTUs were higher in the SUI group (the size and quantity of solid triangles were higher than those of hollow triangles, Fig. 3A). The relative abundance of *Lactobacillus* in the SUI group significantly decreased (FDR < 0.05) (Fig. 3A). At the phylum level, the distribution of the phylum *Bacteroidota* was significantly different between the two groups. Group SUI had a significantly higher relative abundance of *Bacteroidota* (Kruskal-Wallis test, *P* < 0.05), while *Firmicutes* was more abundant in group Non-SUI (Fig. 3B). Further analysis of bacterial abundance differences at the genus level showed the same results as Manhattan plot. Among the top 15 genera in abundance, the distribution of *Lactobacillus*, *Streptococcus*, *Prevotella*, *Finegoldia,* and *Dialister* was significantly different between the two groups (Kruskal-Wallis test, *P* < 0.05). *Lactobacillus* exhibited significant up-regulation in the Non-SUI group, while *Streptococcus*, *Prevotella*, *Finegoldia*, and *Dialister* were significantly up-regulated in the SUI group. Additionally, the average abundance of *Gardnerella* was higher in the SUI group compared to the Non-SUI group (Fig. 3C).

**Fig. 4:**
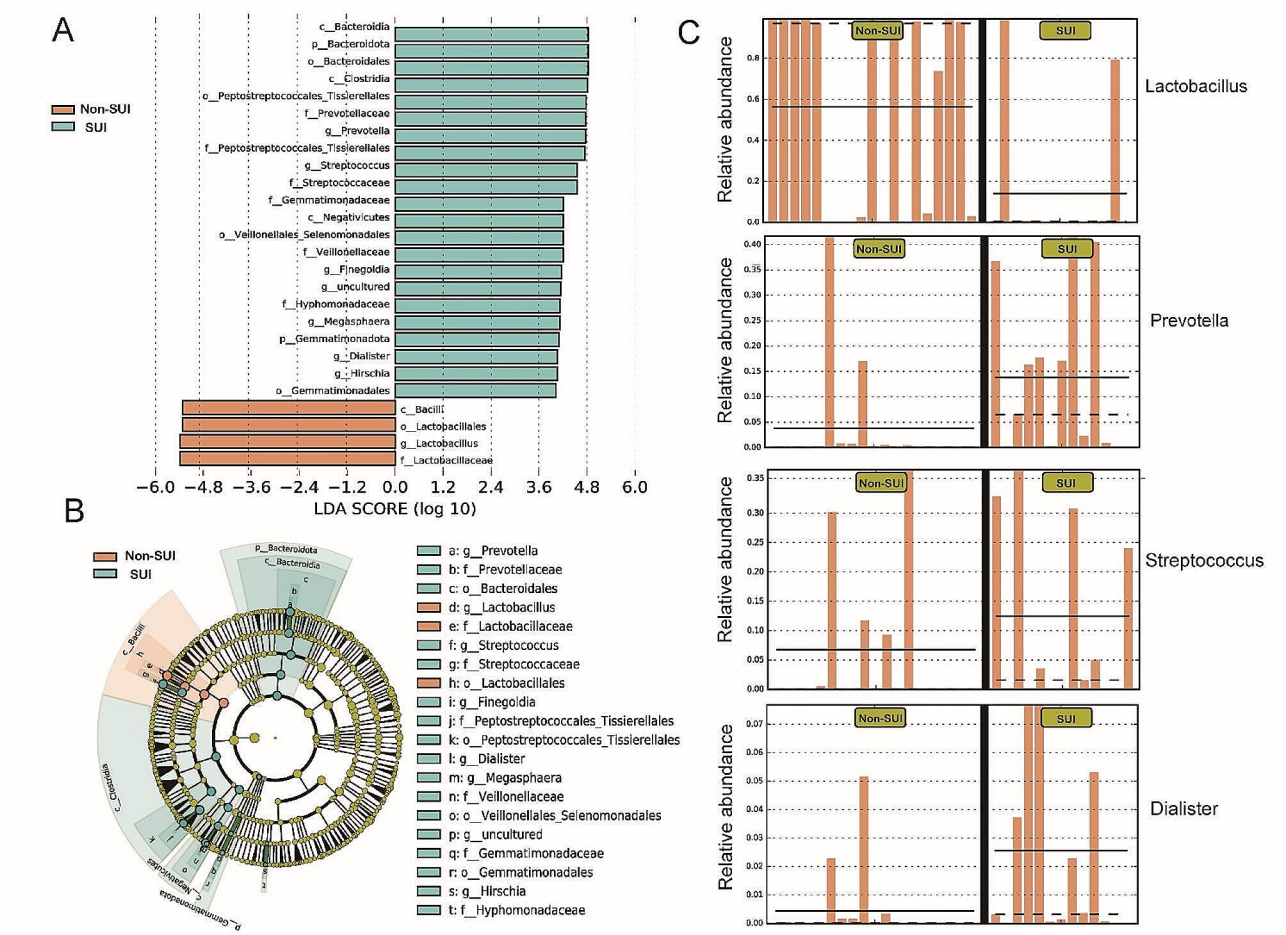
LEfSe analysis of taxonomy with significant differences in abundance among groups. (**A**) The histogram displays the LDA (Linear Discriminant Analysis) values, highlighting biomarkers with statistical differences (LDA > 4) between the two groups, and the length of each bar in the histogram represents the influence of the species with a significant difference. (**B**) Evolutionary branching diagram. Taxonomic levels from phylum to genus are represented by circles that radiate from the center outward. Groups of biomarkers of divergent species are colored, with red nodes denoting microbial taxa, green nodes denoting microbial taxa that play an important role in the SUI group, and purple nodes denoting the Non-SUI group of that species. Species with no significant differences are colored uniformly yellow-green, and the diameter of the circle is proportional to the relative abundance size. The legend on the right shows the names of the species that are denoted by the letters in the illustration. (**C**) Comparison of the abundance of biomarkers in each sample among SUI and Non-SUI.

To better understand the composition characteristics of vaginal microbiota in SUI patients, we defined species with significantly different microbial abundances (*P* < 0.05) between the two groups as inter-group differential markers. The results of LEfSe indicated that *Lactobacillus*, *Prevotella*, *Streptococcus*, *Finegoldia*, *Dialister*, *Hirschia*, and *Megasphaera* were intergroup differential biomarker genera (LDA > 4, *P* < 0.05) (Fig. 4A, B). In addition, we compared the differences in the distribution of the four marker bacteria with higher relative abundance in groups Non-SUI and SUI. Compared to group SUI, group Non-SUI showed a higher relative abundance of *Lactobacillus* and only two samples in group SUI contained high relative abundance of *Lactobacillus*. *Prevotella*, *Streptococcus,* and *Dialister* were more frequent and of higher relative abundance in group SUI (Fig. 4C).

### Network co-occurrence analysis and correlation analysis among vaginal microbiota

**Fig. 5:**
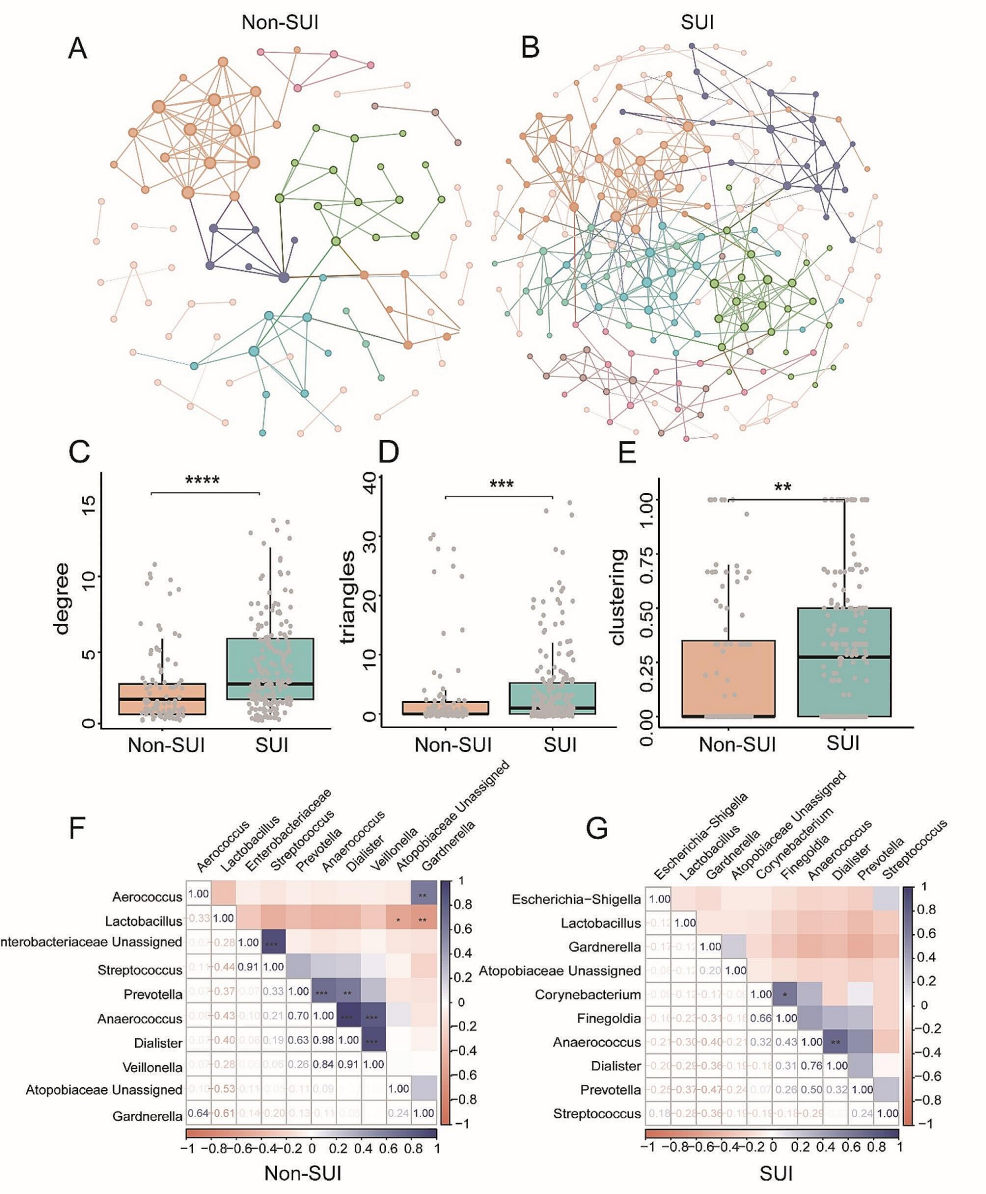
Co-occurrence network analysis and correlation analysis between groups Non-SUI and SUI. (**A**) Network of group Non-SUI. (**B**) Network of group SUI. Comparison of network topology properties among groups, under the same network construction parameters (the absolute value of R is less than 0.6, *P* < 0.05), the more degree, clustering, and triangles appear in the network diagram, the more complex the network is (Wilcoxon test, **P < 0.01; ***P < 0.001; ****P < 0.0001). (**C**) degree (**D**) clustering (**E**) triangles. Correlation analysis, red indicates negative correlation and blue indicates positive correlation. (**F**) Species correlation heatmap of the top 10 abundance bacteria in group Non-SUI at genus level. (**G**) Species correlation heatmap of the top 10 relative abundance bacteria in group SUI at genus level (*P < 0.05; **P < 0.01; ***P < 0.001).

To unravel the relationship between microorganisms, we performed network co-occurrence analysis and correlation analysis. With the same network construction parameters (correlation *r* > 0.6 or *r* < – 0.6, *P* < 0.05), the network in group Non-SUI had 96 nodes and 133 edges (Fig. 5A and Supplementary Table 3) while group SUI had 200 nodes and 409 edges (Fig. 5B). Next, network attribute analysis was performed, and the average degree was 2.771 for group Non-SUI and 4.07 for group SUI. The average degree of group SUI was significantly higher than group Non-SUI (*P* < 0.0001) (Fig. 5C). The number of triangles and the number of sides forming triangles were significantly higher in group SUI than in group Non-SUI (*P* < 0.001) (Fig. 5D). The average clustering coefficient of the SUI was significantly higher than that of Non-SUI (*P* < 0.01) (Fig. 5E). Moreover, a correlation analysis was conducted to examine the interactions among the top 10 most abundant bacterial genera in the Non-SUI and SUI groups, respectively. The results showed that *Gardnerella* and *Lactobacillus* in group Non-SUI showed a significant negative correlation (*P* < 0.01) and a significant positive correlation with *Aerococcus* (*P* < 0.01), *Veillonella*, *Anaerococcus* and *Dialister* showed a significant positive correlation (*P* < 0.001). Additionally, *Dialister*, *Prevotella*, and *Anaerococcus* showed a significant positive correlation (*P* < 0.01, *P* < 0.001), *Anaerococcus* and *Prevotella* showed significant positive correlation (*P* < 0.001) (Fig. 5F). In the SUI group, there was a significant positive correlation between *Dialister* and *Anaerococcus* (*P* < 0.01), as well as between *Finegoldia* and *Corynebacterium* (*P* < 0.01) (Fig. 5G).

## Discussion

It was found that postpartum women with SUI have altered their vaginal microbial community composition, alpha diversity, beta diversity, and co-occurrence network compared to those without SUI. Specifically, the vaginal microbiota of SUI patients was not dominated by *Lactobacillus* but showed an increase in low relative abundance bacteria such as *Gardnerella*, *Streptococcus*, *Prevotella*, *Dialister*, and *Veillonella*. Compared to the Non-SUI group, SUI patients exhibited significantly increased species richness and evenness of the vaginal microbiota, and the microbial composition of the two groups showed distinct clustering patterns. The proportion of vaginal dysbiosis was higher in the SUI group (84.2% vs 42.1%). Additionally, the co-occurrence network of vaginal microbiota in SUI patients exhibited greater complexity.

Microbial homeostasis is important for maintaining vaginal health. When the abundance of *Lactobacillus* in the vaginal environment drops sharply, it indicates dysbiosis of vaginal microbiota [31]. A previous study found no significant correlation between postpartum vaginal microbiota and urinary incontinence [32]. Nevertheless, our results demonstrated a certain degree of correlation between SUI and vaginal microbial imbalance, which may be attributed to differences in sample selection, study design, and geographical variations. Our study found a correlation between SUI and the vaginal microbiota composition in postpartum women, with a highly diverse genus-level vaginal microbiota, notably containing *Gardnerella*, *Streptococcus*, and *Prevotella*, which were associated with BV [33]. In other words, our study revealed a significant decrease in the relative abundance of the predominant probiotic bacteria (*Lactobacillus*) in the vaginal microbiota of patients with SUI, accompanied by an increase in the species and quantity of opportunistic pathogens, which may lead to a weakened ability to maintain internal balance in the vaginal environment. Disruption of the balanced vaginal microbiota, characterized by a shift in microbial communities and the potential development of biofilms, can lead to vaginal dysbiosis, increasing the risk of various infections and negatively impacting women’s reproductive health outcomes [34]. Further studies are needed to establish the causality between vaginal microbial imbalance and disease.

Additionally, research has shown that BV may be associated with urinary incontinence, and women with BV were more likely to report SUI [35]. BV is characterized by a decrease in *Lactobacillus* (the dominant species in a healthy vaginal microbiota) and an increase in anaerobic bacteria such as *Gardnerella*, *Prevotella*, and *Atopobium*, leading to pelvic floor disorders (PFD) [36]. These pathogenic bacteria could cause tissue injury and increased vulnerability to urine incontinence by causing persistent inflammation defined by neutrophil infiltration and immune molecules and cytokines activation [12, 37]. Furthermore, it is concerning that antimicrobial resistance is receiving increasing attention and has become more frequent and widespread in recent decades [38]. This phenomenon poses significant challenges in the effective treatment of infections caused by these pathogenic bacteria. In our study, the vaginal microbiota of SUI patients showed a stronger correlation with BV and a dysbiosis of microbial communities. Most of the vaginal microbiota of SUI patients were no longer dominated by *Lactobacillus* and showed a significant dysbiosis of microbiota compared to controls. NMDS analysis showed a clustering between samples from SUI individuals and healthy controls pattern. These results were consistent with the findings of previous studies on the relationship between BV and SUI [39, 40]. Another explanation for the link between SUI and the vaginal microbiota is hormonal changes during pregnancy and breastfeeding increase a woman’s susceptibility to genitourinary tract infections, potentially affecting the composition of the vaginal microbiota [41]. In addition, childbirth may cause damage to pelvic floor muscles and nerves, leading to pelvic organ prolapse and changes in the composition of vaginal microbiota [42]. Study has shown that the presence of PFD similar to SUI after delivery was associated with the degree of pelvic floor damage, which may trigger an imbalance in the vaginal microbiota, a disruption in the dominance of *Lactobacillus*, and an increase in microbial diversity [32]. Although our study did not specifically analyze this association, we found that patients who experienced SUI also exhibited increased vaginal microbial alpha diversity, consistent with the findings reported in the aforementioned study. However, the specific molecular mechanisms by which urinary incontinence affects the composition and diversity of the vaginal microbial community still need to be further investigated.

Dysbiosis is manifested not only by changes in the abundance and species of microorganisms that make up the community but also by changes in the interactions between microorganisms [43]. In a healthy state, the vaginal microbiota relies on a diverse consortium of *Lactobacillus* species to maintain vaginal health and prevent dysbiosis. Among the important *Lactobacillus* species, *Lactobacillus acidophilus*, *Lactobacillus jensenii*, *Lactobacillus crispatus*, *Lactobacillus iners*, and *Lactobacillus gasseri* have been recognized for their crucial role in promoting probiotic activity through multi-microbial interactions [38]. In pregnant women, *L. crispatus*, *L. iners*, and *L. acidophilus* were found to be the most predominant bacterial *Lactobacillus* species in the vaginal tract [17]. When the healthy vaginal microbiota is disrupted, it is often accompanied by vaginal diseases, miscarriage, premature birth, etc. [44]. . The findings of the species correlation and network co-occurrence analyses indicated that SUI patients had stronger clustering features and a more complex network. In general, microbial communities that exhibit a high degree of cooperation are considered less stable [45, 46]. Our findings suggested a potential association between unstable vaginal microbiota and SUI, but the causal relationship remained unclear.

Certain limitations of the present study should also be considered. Firstly, the study group consisted of only 32 postpartum women, which may limit the generalizability of the results. Secondly, the lack of quantification and identification of probiotic *Lactobacillus* versus opportunistic pathogens detected in this study is a notable limitation. But our findings provide important insights into the relationship between SUI and vaginal microbiota composition and emphasize the potential impact of PFD on the vaginal microbiota. However, the mechanisms underlying the potential association between unstable vaginal microbiota and urinary incontinence, including the directionality of this relationship and the specific molecular mechanisms involved, remain unclear.

## Conclusions

Our findings suggest a potential correlation between stress urinary incontinence and vaginal microbiota composition in postpartum women. Maintaining a healthy vaginal microbiota is crucial for women’s reproductive health. Future research should validate and expand our preliminary findings by conducting more extensive research to address the limitations of this study. Further exploration of the interactions between vaginal microbiota, urine microbiota, and potential host factors is needed to elucidate the potential mechanisms underlying the relationship between vaginal microbiota dysbiosis and SUI.

### Electronic supplementary material

Below is the link to the electronic supplementary material.

Supplementary Material 1

## Data Availability

The raw sequence data reported in this paper have been deposited in the Genome Sequence Archive [47] in National Genomics Data Center [48], China National Center for Bioinformation / Beijing Institute of Genomics, Chinese Academy of Sciences (GSA: CRA011363) that are publicly accessible at https://ngdc.cncb.ac.cn/gsa.

## Abbreviations

SUI: Stress urinary incontinence
Non-SUI: Postpartum women without SUI
NMDS: Non-metric multidimensional scaling
OTU: Operational Taxonomic Units
LDA: Linear Discriminant Analysis
SD: Standard deviation
BV: Bacterial vaginosis
VC: Vulvovaginal candidiasis
AV: Aerobic vaginitis
PFD: Pelvic floor dysfunction
