# Supplementary material for: Effect of stress urinary incontinence on vaginal microbial communities
Source: BMC Microbiol. 2024 Apr 4;24:112. doi: 10.1186/s12866-024-03237-0 (PMC10993610; doi:10.1186/s12866-024-03237-0)
Supplement: Supplementary file 1 — Supplementary Material 1 [file 12866_2024_3237_MOESM1_ESM.docx]

Supplementary Material

Effect of Stress Urinary Incontinence on Vaginal Microbial Communities

**Man Zhang^1†^,** **Yanhua Zhou^2†^, Siqi Yao****^1^, Yiming Zhao^1^,** **Syeda Sundas Batool ^1^, Jing Huang^3^, Li Jiang^2^, Dayu Yan^4^, Wenguang Yan^2*^, Zheng Yu****^1*^**

*** Correspondence:**

Zheng Yu, E-mail: yuzheng@csu.edu.cn

Wenguang Yan, E-mail: ywgpmn@csu.edu.cn

# Supplementary Tables

**Supplementary Table 1** **Clinical characteristics of the study subjects**

| Variables | Stress Urinary incontinence (SUI)  Non-SUI (n = 19) SUI (n = 13) | | *P* |
| --- | --- | --- | --- |
| Age | 33.5 (28-43) | 33.5 (25-42) | 1.00 |
| BMI | 21.43±1.18 | 23.53±2.61 | 0.036 |
| Overweight (BMI > 24) |  |  | 0.038 |
| Yes | 2 (10.52%) | 6 (46.15%) |  |
| No | 17 (89.48%) | 7 (53.85%) |  |
| Gestation count | 2.05±1.22 | 1.76±1.09 | 0.47 |
| Parturition count | 1.57±0.60 | 1.46±0.66 | 0.57 |
| Vaginal Delivery (VD) or cesarean section (CS) |  |  | 0.72 |
| VD | 12 (63.16%) | 7 (53.84%) |  |
| CS | 7 (46.84%) | 6 (46.16%) |  |
| Gain weight during pregnancy (kg) | 12.55±3.55 | 13.76±4.86 | 0.73 |
| Birthweight (kg) | 3.46±0.30 | 3.17±0.73 | 0.52 |

Continuous data were reported by mean ± standard deviation (SD).

Mann-Whitney U test was used to compare the difference of continuous variables between SUI and Non-SUI groups.

Fisher's exact test was used to compare the relationship between categorical variables of two groups.

**Supplementary Table 2** **Binary logstics regression analysis**

|  | B^a^ | Standard Error^b^ | Wald^c^ | P^e^ | OR^f^ |
| --- | --- | --- | --- | --- | --- |
| age | -0.141 | 0.108 | 1.686 | 0.194 | 0.869 |
| SUI (1) | 4.419 | 1.831 | 5.821 | 0.016 | 82.977 |
| Way (1) | 0.306 | 1.228 | 0.062 | 0.803 | 1.358 |
| Gestation | 0.089 | 0.436 | 0.042 | 0.838 | 1.093 |
| Parturition | -0.307 | 1.081 | 0.08 | 0.777 | 0.736 |
| BMI | -0.523 | 0.294 | 3.173 | 0.075 | 0.593 |
| Gain weight | -0.181 | 0.151 | 1.423 | 0.233 | 0.835 |
| Birthweight | -0.767 | 1.844 | 0.173 | 0.677 | 0.464 |

Independent variable: age, SUI, way, Gestation, Parturition, BMI, gain weight, birthweight.

Dependent variable: vaginal microbial dysbiosis

SUI (1): stress urinary incontinence (0 means no stress urinary incontinence)

way (1): Vaginal Delivery (0 means cesarean section)

OR^f^: Reflections where there is an effect of the independent variable on the dependent variable, OR > 1 indicates that the independent variable will promote the occurrence of the dependent variable outcome.

**Supplementary Table 3** **The topological features of network**

|  | Nodes ^a^ | Edges^b^ | Average modularity_class ^c^ | | Average clustering ^d^ | Average triangles ^e^ | Average closnesscentrality ^f^ | Average degree ^g^ | |
| --- | --- | --- | --- | --- | --- | --- | --- | --- | --- |
| Non-SUI  SUI | 96  200 | 133  409 | 15.69  18.07 | 0.22  0.31 | | 2.94  4.35 | 0.51  0.21 | | 2.77  4.09 |

a Number of OTUs with the correlation r > 0.6 or r < –0.6 and statistical significance (*P* < 0.05)

b Number of strong and significant correlations between nodes

c The number of modular structures, modularity indicates that some nodes in the network are more densely connected to each other than to the rest of the network and that their density is significantly higher than the average value of the graph.

d The extent to which nodes are clustered together

e The more the number of triangles formed, the more connected the nodes are in the network

f The node is connected to the whole network, the higher the value, the greater the role played in the network diagram

g The average number of steps along the shortest paths for all possible pairs of network nodes

# Supplementary Figures


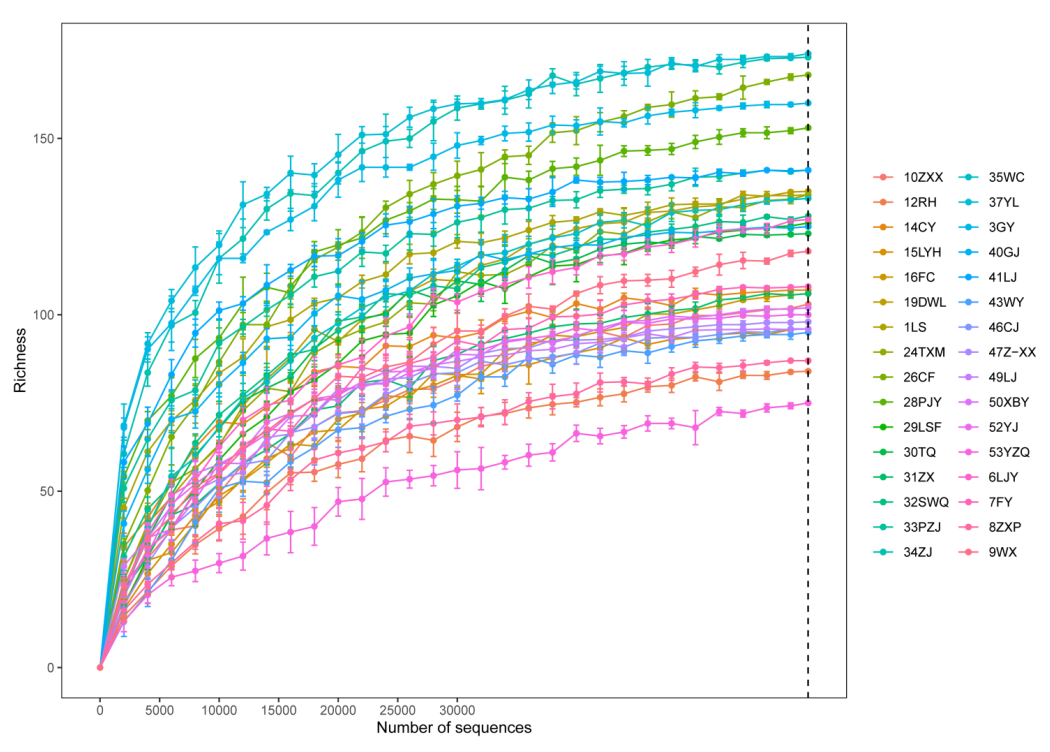


**Supplementary Figure 1.** **Alpha dilution curve for all samples.** As the sequencing depth increases, the dilution curve of bacterial Operational Taxonomic Units (OTUs) detected from the vaginal microbiota reaches the saturation stage. Each error line represents the standard error.
